# Genomic landscape of metastatic breast cancers in young adults: a liquid biopsy analysis of women aged 20–40 years

**DOI:** 10.1016/j.breast.2025.104690

**Published:** 2026-01-02

**Authors:** Ernest Diab, Cyril Roussel-Simonin, Federica Giugliano, Julia Dixon-Douglas, Alessandra Spata, Martina Pagliuca, Lauriane Minot, Alexandre Xu-Vuillard, Fernanda Mosele, Thomas Grinda, Alessandro Viansone, Chayma Bousrih, Jean Zeghondy, Tarek Ben Ahmed, Claudio Nicotra, Arnaud Bayle, Antoine Italiano, Suzette Delaloge, Barbara Pistilli, Fabrice André, Joana Ribeiro, Elie Rassy

**Affiliations:** a Department of Cancer Medicine, Gustave Roussy, Villejuif, France; b Department of Oncology and Hemato-Oncology, University of Milan, Milan, Italy; c Université Paris-Saclay, Gustave Roussy, Inserm U981, 94805, Villejuif, France; d Sir Peter MacCallum Department of Medical Oncology, University of Melbourne, 305 Grattan Street, Parkville, Australia; e Gustave Roussy, DITEP, Gustave Roussy, Villejuif, France; f Bureau Biostatistique et Epidémiologie, Gustave Roussy, Université Paris-Saclay, Villejuif, France; g Oncostat U1018, Inserm, Université Paris-Saclay, Equipe labellisée Ligue Contre le Cancer, Villejuif, France; h Department of Medical Oncology, Institut Bergonié, University of Bordeaux, INSERM, Unité ACTION U1218, Bordeaux, France; i Université Paris-Saclay, Gustave Roussy, INSERM, Gustave Roussy, U1279, Villejuif, France

## Abstract

**Introduction:**

Breast cancer in young adults (YA) aged 20–40 years has distinct clinical and biological traits compared with older patients. This study evaluated the genomic landscape of metastatic breast cancers (MBC) among YA.

**Methods:**

Patients with MBC enrolled in the STING molecular profile platform (NCT04932525) between 2021 and May 2023 were included. Clinical and genomic features were analyzed by age (≤40 vs > 40 years). Tumor profiling used the FoundationOne Liquid CDx assay (324 genes) at baseline or later in the disease course. Variant frequencies were compared across age groups.

**Results:**

Of 432 eligible patients, 68 (16 %) were YA. Among 37 YA with hormone receptor positive (HR+) BC, frequent alterations included *TP53* (39 %), *ESR1* (27 %), *PIK3CA* (25 %), *FGFR3* (18 %), *FGFR4* (18 %), *FGFR19* (18 %), *CCND1* (18 %). Compared with older patients, YA with HR + tumors had fewer *RB1* (7 % vs 8 %; *p* = 0.03) and *PIK3CA* (25 % vs 31 %; *p* = 0.03) alterations. Among 28 YA with triple negative BC, the most common alterations were *TP53* (100 %), *PTEN* (26 %), *BRCA1* (22 %), *RB1* (17 %). *PTEN* mutations were more frequent among YA with TNBC than older patients (26 % vs 8 %; *p* = 0.009). Tiers I-III genomic alterations according to the ESMO scale of clinical actionability (ESCAT) were identified in 54 YA (79 %), including 48 tiers I-II alterations comprising *ESR1* (n = 12), *gBRCA1/2* (n = 11), *PIK3CA* (n = 13).

**Conclusions:**

ESCAT tiers I-III alterations were reported in 79 % YA with MBC which supports the role of molecular profiling in YA. The differences detected in the genomic profiles of YA with BC and older patients may allude to potential different underlying disease biology.

## Highlights

• Young adults with metastatic breast cancer present distinct genomic profiles in comparison to older patients.
• In young adults with metatatic breast cancer, 79% present actionable ESCAT I-IV and ESCAT I-III genomic alterations.
• Cell cycle and DNA damage response pathway alterations were more common in young adults.
• These findings underline the potential impact of liquid biopsy in young adults for a genome-guided personalized therapy.

## 1 Introduction

Cancer in young adults (YA), usually referred to as ‘’early-onset cancer’’, is an emerging public health burden [1]. The incidence of cancers in YA has increased by nearly 80 % from 1990 to 2019, with early-onset cancer-related deaths also increasing by approximately 30 % [2]. While mortality rates and incidence of all cancers combined are significantly lower in younger adults than in older individuals, cancers affecting YA have important economic and social consequences and can lead to a higher number of person-years of life lost compared to those diagnosed later in life [[3], [4]]^.^ There is no universally accepted biological definition of early-onset cancers and the cutoff age for YA with cancer has been inconsistent throughout the literature. For breast cancer (BC), the commonly used 50-year threshold aligns with established screening guidelines and coincides with significant biological transitions, particularly in women. On the other hand, having a threshold at 40 years would guide studies to focus on specific issues such as fertility, family planning and economic dependence [[5], [6], [7], [8]]. Moreover, from an epidemiological standpoint focusing on YA with cancer under the age of 40 years would better identify biological abnormalities as the increasing incidence is mostly observed in this age group [9].

Breast cancer is the most common cancer among YA and the leading cause of cancer death in young women [10]. In 2022, BC accounted for approximately 245,563 cases and 48,272 deaths annually worldwide among patients under the age of 40[11]. Over the next 20 years, projections estimate an 11.6 % increase in the incidence of BC among YA [12]. Active efforts are ongoing to study the potential reasons for this increased incidence and to develop dedicated care pathways [13]. In this context, refining the specific genomic characteristics of BC in YA is of interest given their more aggressive clinical and prognostic features compared to older adults [[14], [15], [16], [17], [18], [19], [20]]. The clinical benefits of using next-generation sequencing (NGS) in patients with metastatic BC (MBC) have led to many genomically targeted therapy approvals such as PI3K/AKT inhibitors for *PIK3CA/AKT/PTEN*-mutated tumors, PARP inhibitors for *BRCA1/2* mutations and selective estrogen receptor downregulators (SERDs) for *ESR1* mutations [[21], [22], [23], [24], [25]]. In 2024, the ESMO Precision Oncology Working Group recommended to perform tumor profiling in daily clinical practice to cover of seven BC specific and six tumor-agnostic actionable genomic alterations (Supplementary Table 1) [26]. In this paper, we compared genomic profiles from circulating tumour DNA (ctDNA) in patients with MBC aged 40 years and younger with genomic profiles of patients older than 40 years.

## 2 Materials and methods

### 2.1 Patients

This retrospective study used individual-participant data from women with MBC treated at Gustave Roussy Cancer Center and having undergone a liquid biopsy for somatic molecular profiling within the cross-sectional STING platform (NCT04932525). Liquid biopsy was performed at any point during the course of their metastatic disease, between 2021 and May 2023. The electronic health records of the eligible patients were used to collect clinical and pathological data. Patient's age was calculated at the time of first metastatic disease. Therefore patients diagnosed with MBC before age 40 and sampled after turning 40 were included in the YA cohort. For patients who underwent multiple liquid biopsies, only the results of the first biopsy were considered. Eligible patients were categorized according to the histological BC subtypes as determined at the time of the tumor biopsy of MBC. Hormone receptor positivity was defined as immunohistochemical [IHC] estrogen or progesterone receptor expression ≥10 %, and triple negative was defined was defined as ER < 10 % and PR < 10 % and HER2 non-overexpressed and non-amplified. HER2 amplification was defined according to ASCO CAP guidelines. Patients with missing clinical data and without an available molecular profile were excluded. All participants have signed a written informed consent document for their participating in the STING study.

### 2.2 Molecular profiling

In the STING molecular profile platform, liquid biopsy analysis was performed on plasma samples collected using the FoundationOne® Liquid CDx (Foundation Medicine, Inc; Cambridge, MA) assay covering 324 genes and offering a limit of detection as low as 0.1 % variant allele frequency (VAF) for single nucleotide variants (SNVs). Only genomic alterations classified as pathogenic and present in the clinical report generated by Foundation Medicine were retained for this study. The test also reports blood tumor mutational burden (bTMB), microsatellite stability status (MSS) and tumor fractions (TF) values. Blood TMB was estimated from the number of synonymous and non-synonymous single nucleotide variants (SNVs) and insertions and deletions (indels) per area of coding genome sampled, after the removal of known and likely oncogenic driver events and germline single nucleotide polymorphisms. Samples were classified as high-TMB if ≥ 10 mutations per megabase (Mut/Mb) were observed. Circulating tumor fraction was estimated based on a normalized coverage level across the genome and bTMB was calculated based on all the mutations identified with a VAF >0.5 %. The identified genomic alterations were scored using the ESMO Scale for Clinical Actionability of Molecular Targets (ESCAT), a tool that helps clinicians to select the most appropriate targeted therapies based on the highest expected benefit for the patient (Supplementary Table S1). Each genomic alteration has an ESCAT score reported as ‘’tier’’ (I, II, III, IV, V, X). Tier I indicates that the alteration–drug match is associated with improved outcomes in clinical trials, II shows antitumor activity with uncertain benefit, III is suspected benefit based on other tumors or related alterations, IV has preclinical evidence, V shows objective response without meaningful benefit and X lacks evidence of actionability [27]. We also excluded clonal hematopoiesis of indeterminate potential (CHIP) mutation from the identified genomic alterations.

### 2.3 Statistics

Descriptive statistics, reporting proportions for qualitative variables and median with range for continuous variables, were used to summarize patient and tumor characteristics. Median time between metastatic disease diagnosis and liquid biopsy was estimated by Kaplan Meier methodology. Given that the objective of this study was to identify the molecular characteristics of YA with MBC, in comparison to the older patients, we compared the somatic molecular profile of patients with MBC among YA ≤ 40 years in comparison to patients >40 years enrolled in the STING study. We compared the number (proportions) of genomic alterations, microsatellite instability (MSI) and TMB in the two groups. We also did a descriptive analysis of pathway alterations without performing formal statistical testing. For each gene, mutation frequency was compared between age groups using either Fisher's exact test or Pearson's chi-squared test, as needed. Mutation frequencies and corresponding p-values were visualized using barplots stratified by age group.

## 3 Results

At the time of data curation in December 2024, a total of 432 patients with MBC have had liquid biopsies performed within the STING study. 68 (16 %) patients were YA (≤40 years old) and 364 (84 %) were “older” adults (>40). In the YA group, 50 patients were aged 40 years or younger at the time of liquid biopsy sampling whereas 18 patients had their liquid biopsy performed after 40 years of age. Demographic and baseline characteristics of YA are shown in Table 1. Among YA, the majority (n = 37) had HR+, 3 had HER2-Positive and the rest (n = 28) had TNBC. Molecular profiling was performed at the metastatic stage, with 19 patients (28 %) having liquid biopsy sampling at baseline (prior or concurrent to first line therapy), 13 patients (19 %) after first-line therapy, 9 patients (13 %) after second-line therapy and 27 patients (40 %) after third-line therapy or beyond. The median interval time between diagnosis of metastatic disease and liquid biopsy was 19.7 months in both young and older cohort (95 % confidence interval [CI]: 16.8–22.7).

**Table 1:** Demographic and clinical characteristics.

Characteristics		Number of patients (%) Total 68 patients
**Age at MBC diagnosis – year**	20 to 29	5 (7.4 %)
	30 to 34	22 (32.4 %)
	35 to 40	41 (60.2 %)
**Age at ctDNA – year**	20 to 29	2 (3.0 %)
	30 to 34	14 (20.6 %)
	35 to 40	34 (50 %)
	>40	18 (26.4 %)
**Histological subtypes**	TNBC	28 (41.2 %)
	HR + BC	37 (54.4 %)
	HER2-Positive	3 (4.4 %)
g*BRCA1* mutation^a^	HR + BC	0
	TNBC	4
g*BRCA2* mutation^a^	HR + BC	5
	TNBC	2
**Visceral metastases**	TNBC	18 (26.5 %)
	HR + BC	19 (28.0 %)
	HER2-Positive	3 (4.4 %)
**Metastatic status**	De novo	19 (27.9 %)
	Metachronous	49 (72.1 %)
**Lines of therapy after metastatic disease at liquid biopsy sampling – no.**	At baseline or frontline therapy	19 (27.9 %)
	After first line therapy	13 (19.0 %)
	After second line therapy	9 (13.2 %)
	After third line therapy and beyond	27 (39.9 %)

Abbreviations: g*BRCA*: germline *BRCA*; HR + BC: Hormone receptor positive breast cancer; TNBC: Triple negative breast cancer.

a 1 patient had the analysis but died before unveiling the result.

We also reported the germline mutational status of *BRCA1/2* genes among the 68 YA from their electronic health records. Eleven patients were found to carry *BRCA1/2* germline mutation, one patient with a germline *TP53* mutation was identified, while 53 had no detectable germline mutations. Four had no available germline genetic results.

**Fig. 1:**
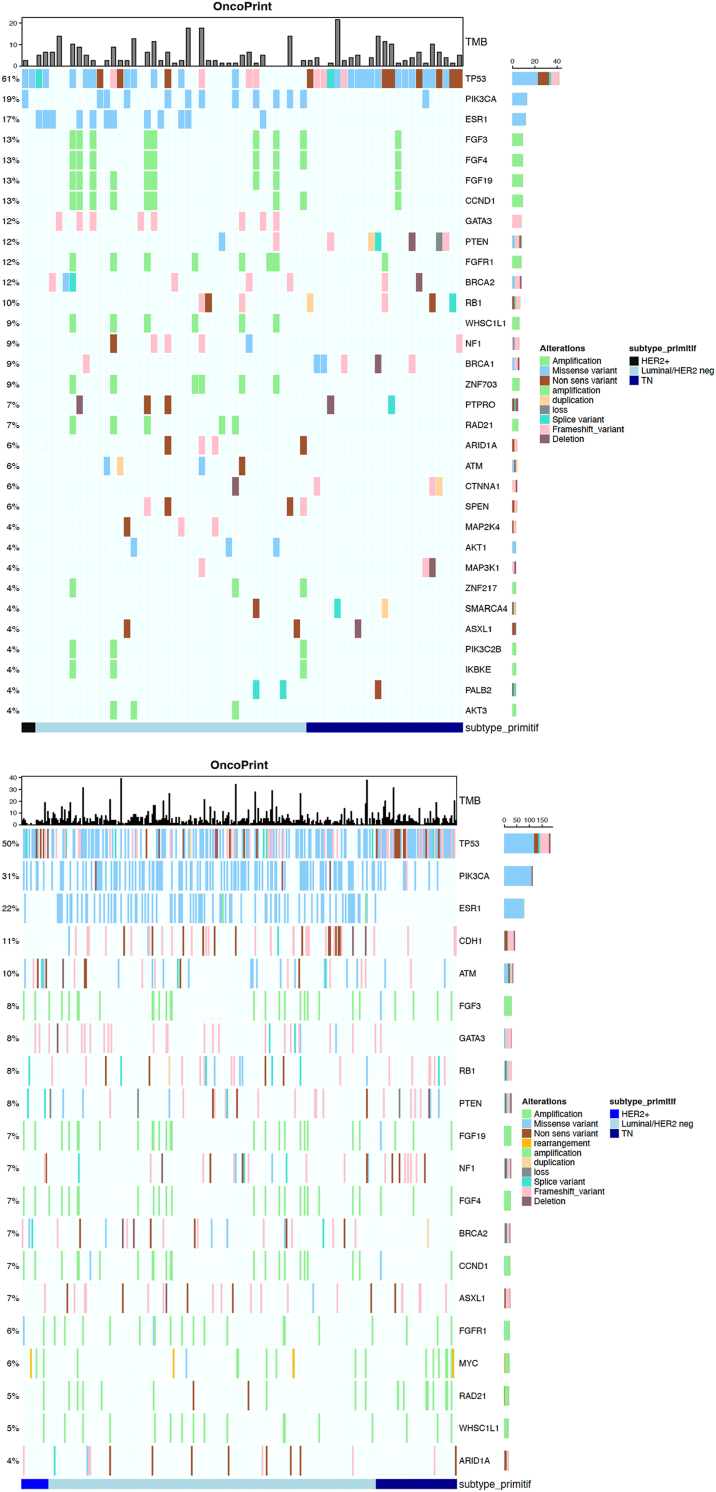
Oncoplot of genomic alterations in MBC among YA (A) and patients (B) older than 40 years. Legend Fig. 1A: In YA the most frequently altered genes were TP53, PIK3CA and ESR1. Legend Fig. 1B: In older patients, TP53, PIK3CA, and ESR1 were most commonly altered. Abbreviations: MBC: metastatic breast cancer; YA: young adult.

**Fig. 2:**
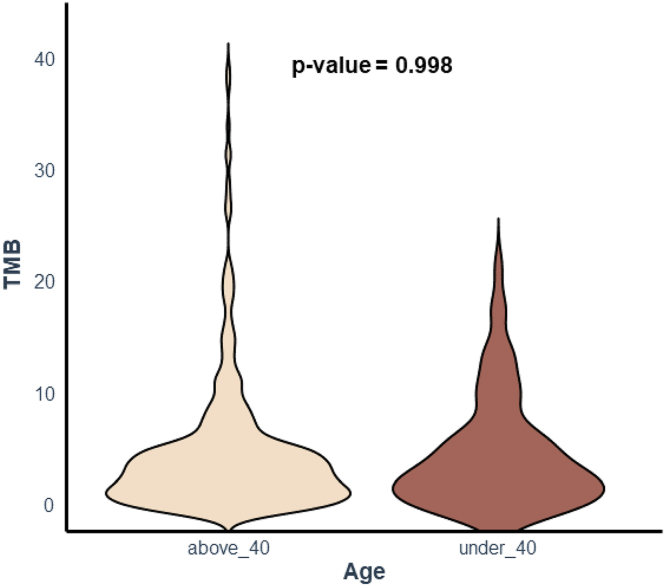
Violin plot of bTMB according to age group. Legend Fig. 2: Median bTMB was lower in YA compared to older patients.

**Fig. 3:**
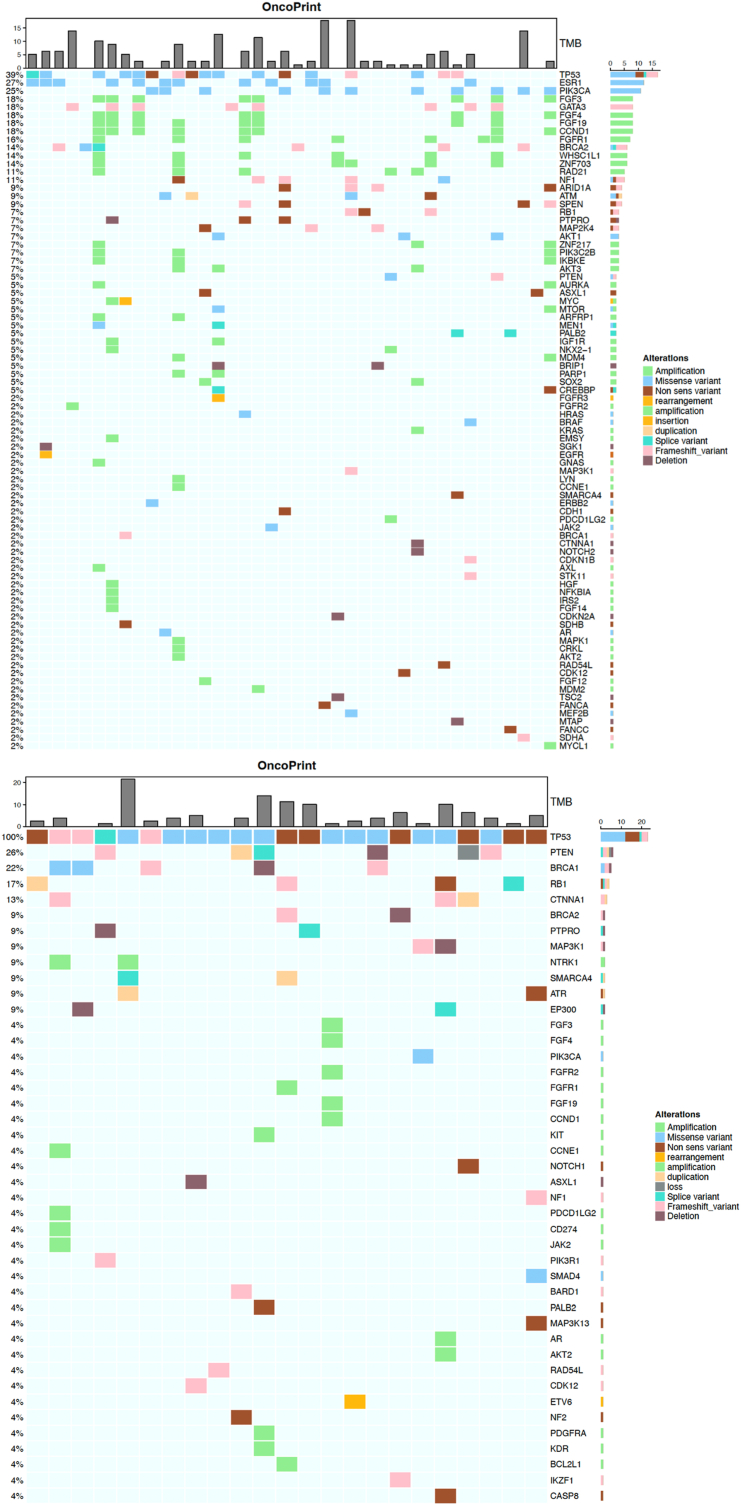
Oncoplots of genomic alterations in MBC among YA with HR+ (A) and triple negative BC (B). Legend Fig. 3A: In HR + MBC the most frequently mutated genes were TP53, ESR1 and PIK3CA. Legend Fig. 3B: In triple-negative MBC frequent alterations were observed in TP53, PTEN, and BRCA1. Abbreviations: HR+: hormone receptor positive; MBC: metastatic breast cancer; TNBC: triple-negative breast cancer; YA: young adult.

A total of 1343 pathogenic genomic alterations were identified. Altered genes in YA and older patients are presented in Fig. 1A and B. Overall, *TP53* (61 % vs 50 %) represented the most altered gene in both groups. *PIK3CA* was mutated in 19 % and 31 % of YA and older patients, respectively (*p* = 0.021). *BRCA1* (9 % vs 2 %, *p* = 0.022) and *SMARCA4* (4 % vs <1 %, *p* = 0.023) mutations were more frequent in YA. Blood TMB was determined for all patients. Median bTMB was 2.53 Mut/Mb in YA, versus 3.79 in older patients, and 8 YA cases (12 %) presented a bTMB ≥10 Mut/Mb, compared with 48 cases (13 %) having a bTMB of ≥10 Mut/Mb in older adults *(p* = 0.998*)* (Fig. 2). Stratifying for BC subtype in YA (Fig. 3), the most frequently altered genes consisted of *TP53* (39 %), *ESR1* (27 %), *PIK3CA* (25 %), *FGFR3* (18 %), *FGRF4* (18 %), *FGFR19* (18 %), *CCND1* (18 %) for HR + BC and *TP53* (100 %), *PTEN* (26 %), *BRCA1* (22 %), *RB1* (17 %), for triple negative BC. In HR + BC, among YA, *RB1* (7 % vs 8 %; *p* = 0.03) and *PIK3CA* (25 % vs 31 %; *p* = 0.03) mutations were less frequent in comparison to > 40 years old patients. In triple negative BC, YA patients more commonly had *PTEN* mutations compared to older patients (26 % vs 8 %; *p* = 0.009) and less commonly *PIK3CA* mutations (4 vs 31 %; *p* < 0.05).

**Fig. 4:**
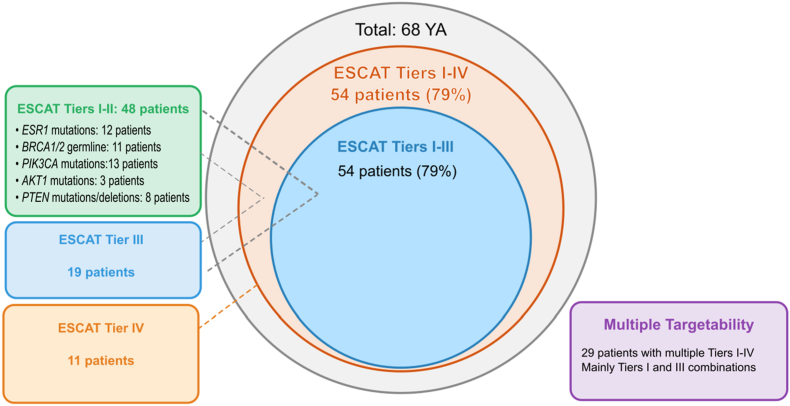
ESCAT targetability distribution in YA. Legend Fig. 4: Tier I-II alterations were the most frequently observed, including ESR1, BRCA1/2, PIK3CA, AKT1 and PTEN alterations. Eight YA had bTMB ≥10 Mut/Mb. Abbreviations: ESCAT: ESMO Scale for Clinical Actionability of molecular Targets; MBC: metastatic breast cancer; YA: young adult.

Next, we conducted an analysis of genomic alterations according to ESCAT (Fig. 4). *ERBB2* amplification, germline *BRCA1/2* mutations, *PIK3CA* mutations, and *ESR1* mutations were mainly classified as tier I-A according to ESCAT in advanced BC whereas agnostic genomic alterations (*NTRK1/2/3* fusions, MSI-H/dMMR, *RET* fusions, *BRAF* mutations, *FGFR1/2/3* fusions or mutations and TMB-H) were classified as tier I-C according to ESCAT's list of tumor-agnostic genomic alterations. Tiers I-III and I-IV genomic alterations according to ESCAT were identified in 54 YA (79 %). Forty-eight YA had ESCAT tiers I-II including 12 with *ESR1* mutation, 11 with *BRCA1/2* germline pathogenic variant, 13 with *PIK3CA* hotspot mutation, 3 with *AKT1* mutation and 8 with *PTEN* mutation or deletion. Nineteen YA had ESCAT tier III alterations including *EGFR*, *HRAS*, *MTAP*, *TP53*. Eleven YA had ESCAT tier IV alterations. Twenty-nine YA had multiple ESCAT alterations with most having combinations of tiers I and III. Table 2 summarizes the identified genomic alterations and their corresponding characteristics, and Supplementary Table 1 reports on the genomic alterations in YA along with the corresponding activity level. Among the 48 patients with ESCAT tier I alterations, eleven patients with *BRCA1/2* germline pathogenic variants received PARP inhibitors, as germline testing is systematically performed at initial consultation. Among patients with *ESR1* or *PIK3CA* alterations only a subset received targeted therapies. This variability was due to the timing of the liquid biopsy as several samples were obtained beyond the first line setting when patients were no longer eligible for these treatments.

**Table 2:** Comprehensive overview of genetic alterations identified, including number of affected patients and associated functional activity.

Gene	Genetic alterations	Number of patients	Activity Level	Notes
*ESR1*	Y537S	3	Strong	Single alteration
*ESR1*	D538G	2	Moderate	Single alteration
*ESR1*	E380Q + H356D + D538G + L536H + Y537S	1	Strong + Moderate + Weak + Modest	Multiple alterations
*ESR1*	Y537N + Y537S	1	Strong	Dual Y537 variants
*ESR1*	D538G + Y537S	1	Moderate + Strong	Multiple alterations
*ESR1*	L536H + D538G	2	Modest + Moderate	Multiple alterations
*ESR1*	P535_L536insP + E538G	1	Unknown	Multiple alterations
*ESR1*	L536P + H356D	1	Modest	Multiple alterations
*PIK3CA*	N345K + H1047R + E542K + E545K	1	–	Multiple hotspot alterations
*PIK3CA*	E542K + amp	2	–	Helical domain alteration + amplification
*PIK3CA*	E545K	2	–	Helical domain alteration
*PIK3CA*	H1047R	2	–	Kinase domain alteration
*PIK3CA*	V105_K107>D	1	–	Complex rearrangement
*PIK3CA*	E453K + amp	1	–	Helical domain alteration + amplification
*PIK3CA*	E542K	2	–	Helical domain alteration
*PIK3CA*	K111E	1	–	Protein's linker region alteration
*PIK3CA*	R88K	1	–	Adaptor-binding domain alteration
*BRCA1*	Various mutations	6	–	4 germline BRCA1 mutations
*BRCA2*	Various mutations	8	–	7 germline BRCA2 mutations
*TP53 + BRCA1*	Co-occurring mutations	6	–	Dual tumor suppressors
*TP53 + BRCA2*	Co-occurring mutations	4	–	Dual tumor suppressors

To explore whether BC in YA is biologically distinct from that diagnosed in older patients we conducted an exploratory analysis to identify pathway signaling differences between age groups. Cell cycle (75 vs 62 %) and DNA damage response (52 vs 40 %) pathway alterations were more common in YA whereas growth factor signaling such as PIK3-AKT-mTOR (36 vs 72 %), FGF/FGFR (19 vs 27 %) and MAPK (20 vs 25 %) pathway alterations were more common in the pts >40 years.

## 4 Discussion

In line with the active efforts for a comprehensive understanding of the molecular biology of BC in YA [[28], [29], [30], [31], [32], [33]], the present work evaluated the genomic landscape of MBC using liquid biopsy and its clinical implications in YA. Our cohort included a majority of YA with HR + MBC (37 patients) and 28 with TNBC. Somatic molecular profiling in YA with HR + MBC showed an enrichment in several genomic alterations, some of which are clinically relevant in current practice such as *ESR1* and *PIK3CA*, which complements previous publications and international guidelines [26,28]. The other alterations in HR + BC and the detected ones in TNBC may not be targetable with currently approved therapies but present opportunities for investigation in clinical trials or as part of tumor-agnostic treatment approaches [34].

For patients with HR + MBC, the cornerstone of the first-line therapy is the combination of CDK4/6 inhibitors and endocrine therapy. Germline *BRCA1/2* mutations [35], detected in 11 YA (16 %), may be associated with resistance to CDK4/6 inhibitors. *RB1* co-deletion with *BRCA2*, due to synteny on chromosome 13q, may explain reduced efficacy of CDK4/6 inhibitors in *BRCA2* mutant cancers [36]. *BRCA1* loss, on the other hand, has been linked to endocrine resistance through estrogen receptor α downregulation [37] and PI3K/AKT pathway activation [38,39]. Additionally, the CDK4/6-cyclin D complex typically activates p21, a mechanism that may be disrupted in *BRCA1*-mutant cells, potentially leading to resistance to CDK4/6 inhibitors [40]. *ESR1* alterations, established drivers to endocrine therapy resistance [41], were identified in twelve YA patients. *ESR1* alterations have distinct patterns of activation with three mutation sites having strong activity (*Y537S*, *Y537N* and *Y537C*), two moderate activity (*D538G* and *S463P*) and five modest or weak activity (*L536 R/H/P/Q* and *E380Q*) [42]. In our study, 6 patients had an alteration involving a strong activity site (*ESR1 Y537S* or *ESR1 Y537N*), 6 patients on a moderate activity site (*ESR1 D538G*) and 4 patients on a modest or weak activity site (*ESR1 L536H* and *ESR1 E380Q*). *ESR1* mutations, namely *ESR1 Y537S* and *ESR1 D538G* mutations, were also associated with worse prognosis in patients treated with CDK4/6 inhibitors [[43], [44], [45]]. However, patients harboring *ESR1* mutations may still derive benefit from CDK4/6i when combined with SERDs rather than AI [46]. This matter was addressed in the PADA-1 and SERENA-6 trials. Investigators found in both trials that patients receiving first-line therapy with an aromatase inhibitor (AI) plus CDK4/6i, switching from AI to SERDs (fulvestrant in PADA-1 and camizestrant in SERENA-6) at *ESR1* mutations detection before tumor progression while continuing CDK4/6 inhibition, resulted in significantly longer PFS compared to continuing AI (11.9 (95 % CI: 9.1–13.6) vs 5.7 months (95 % CI: 3.9–7.5)) in PADA-1 (HR 0.61, 95 % CI: 0.43–0.86, *p* = 0.0040) and 16.0 (95 % CI: 12.7–18.2) vs 9.2 months (95 % CI: 7.2–9.5) in SERENA-6 (HR 0.44, 95 % CI [0.31–0.60]; *p* < 0.0001)) [47,48]. These findings suggest that targeting resistance before tumor progression while maintaining CDK4/6 inhibition is a valid treatment option. *PIK3CA* mutations, known to drive endocrine resistance and worse clinical outcome, were present in 13 YA in our study [[49], [50], [51]]. PI3K inhibitor monotherapy has low response rates, yet in combination with endocrine therapy improvements in survival outcomes have been achieved in HR + MBC with *PIK3CA* alterations [49]. One patient in our study had four distinct alterations in the *PIK3CA* gene, including hotspot substitutions in both the helical (*E542K and E545K*) and kinase (*H1047R*) domains, patterns associated with enhanced PI3K signaling and therapeutic sensitivity to PI3K inhibitors. The remaining patients predominantly had helical domain mutations, with four having the *PIK3CA E542K* variant and two displaying the *PIK3CA E545K* variant. These alterations are in line with a large-scale analysis of 6338 BC samples reporting five recurrent *PIK3CA* alterations, *H1047R* (35 %), *E545K* (17 %), *E542K* (11 %), *N345K* (6 %), and *H1047L* (4 %), accounting for 73 % of all alterations [52]. Prior data show that up to 12 % of BC cases have multiple *PIK3CA* alterations, which enhance PI3K pathway signaling and promote cellular growth more than single alterations. In vivo studies suggest that tumors with double *PIK3CA* alterations have greater sensitivity to PI3K inhibition [52,53].

In metastatic TNBC, YA were more commonly *PTEN* mutated and less commonly *PIK3CA* mutated. Despite the low incidence of *PIK3CA* mutations in YA with TNBC, *PIK3CA* alterations are enriched in older patients with androgen receptor positive TNBC subtypes [54]. While up to 35 % of TNBCs have alterations in *PIK3CA/AKT1/PTEN, AKT* pathway inhibitors have failed to improve survival in this population [55,56]. *BRCA1/2* somatic and germline mutations were reported in 17.9 % and 7.1 % of YA with metastatic TNBC, respectively; *BRCA1* was one of the most altered genes in YA with metastatic TNBC. Beyond predicting PARP inhibitor sensitivity, *BRCA1/2* alterations affect the tumor microenvironment, resulting in increased tumor-infiltrating lymphocyte density and altered T-cell function [[57], [58], [59], [60]].

In addition to subtype alterations of interest, we also assessed agnostic indications covered by liquid biopsy and found eight YA with bTMB ≥10 Mut/Mb. Studies have also focused on using TMB in liquid biopsy with different number of mutational cut-offs [61,62]. TMB has also been associated with increased rates of pathological complete response to chemotherapy as a single agent or in combination with immunotherapy in early-stage TNBC [63,64]. Regarding the tumor agnostic approved indications, their prevalence may have been underestimated in our population due to the inherent limitations of liquid biopsy in detecting gene fusions.

Our findings should be interpreted considering several limitations related to study design, cohort composition and the inherent constraints of ctDNA-based profiling. This study was conducted on a small sample size and was cross-sectional in design, but addresses the important topic of YA with BC, a population that clinicians will encounter with increasing frequency in daily practice [65]. While this study focused on the genomic landscape of YA, comparative analyses of patient characteristics, ESCAT scores, and survival with non-YA patients would provide valuable insights. However, the heterogeneity of the identified mutations would have resulted in small cohort comparisons. Therefore, a descriptive approach specifically to YA was used to evaluate the input of comprehensive profiling in YA with BC. Another limitation resides in performing liquid biopsies mainly after first line or prior therapy, thus excluding long responders to therapy and those with a non-contributive molecular profile. Most targetable alterations are not acquired thus repeating molecular profiling is not currently recommended in patients whose initial samples meet quality assurance standards. The use of DNA-based assays can miss gene fusions if the genomic breakpoints fall within regions not adequately covered by the panel. Additionally, because DNA sequencing only detects breakpoints rather than the fusion transcripts, fusions can be undetected without knowledge of the gene's genomic architecture [66].

## 5 Conclusions and perspectives

Pending validation in a larger cohort, our findings revealed differences in the genomic profiles of YA with BC and older patients which may allude to potential different underlying disease biology. More importantly, 79 % of YA with MBC present actionable genomic alterations classified as ESCAT tiers I-IV, highlighting the role of comprehensive molecular profiling in guiding therapeutic decision-making [67]. While genomic profiling technologies have become increasingly sophisticated and accessible, significant implementation barriers persist in translating these advances into routine clinical practice. The complexity of integrating molecular testing into standard care pathways, interpreting results with appropriate expertise and frameworks, and ensuring timely turnaround times remain substantial challenges.

## CRediT authorship contribution statement

**Ernest Diab:** Writing – original draft, Methodology, Conceptualization. **Cyril Roussel-Simonin:** Methodology, Data curation. **Federica Giugliano:** Data curation. **Julia Dixon-Douglas:** Data curation. **Alessandra Spata:** Data curation. **Martina Pagliuca:** Data curation. **Lauriane Minot:** Writing – review & editing. **Alexandre Xu-Vuillard:** Data curation. **Fernanda Mosele:** Data curation. **Thomas Grinda:** Supervision, Data curation. **Alessandro Viansone:** Writing – review & editing. **Chayma Bousrih:** Writing – review & editing. **Jean Zeghondy:** Writing – review & editing. **Tarek Ben Ahmed:** Writing – review & editing. **Claudio Nicotra:** Writing – review & editing. **Arnaud Bayle:** Writing – review & editing. **Antoine Italiano:** Writing – review & editing. **Suzette Delaloge:** Writing – review & editing. **Barbara Pistilli:** Writing – review & editing. **Fabrice André:** Writing – review & editing. **Joana Ribeiro:** Writing – review & editing. **Elie Rassy:** Writing – review & editing, Validation, Supervision, Data curation, Conceptualization.

## Declaration of competing interest

JDD has received travel and accommodation support for conferences from Novartis, MSD and Pierre Fabre and has received an honorarium from Gilead Life Sciences. MP has received travel grants from Pfizer and Gilead. FM has received consultant fees from Novartis and Pegascy. TG has received travel fees from AstraZeneca, Gilead, and Pfizer; has served in a consulting/advisory role for AstraZeneca, Pfizer, MSD, and Cancerologie-Pratique; has received research funding from Amgen; and has received a personal grant from the Philippe Foundation. AV has received honoraria from Seagen; has served in a consulting/advisory role for Seagen; has served on the speakers’ bureau for AstraZeneca/Daiichi Sankyo; has received research funding from Pfizer; has provided expert testimony for Seagen; and has received travel, accommodations, and expenses support from Eisai Europe. CB has received travel, accommodations, and expenses support from Roche, Novartis, Lilly, Amgen, and Pfizer. JZ has received an institutional research grant from Menarini; has received travel and expenses support from Novartis, Eli Lilly, Pfizer, MSD, and Roche; and has received institutional honoraria from Eli Lilly and Ipsen. AB has received consulting fees from Sanofi (OncoCollective advisory board); has received honoraria from Roche (oral presentation); and has received support for attending ASCO 2023 from Pfizer. AI has served on advisory boards for Bayer, Daiichi Sankyo, Epizyme, Lilly, Novartis, Roche, and SpringWorks, and has received research funding from AstraZeneca, Bayer, Chugai, Merck, MSD, Novartis, and PharmaMar. SD has received grants and non-financial support from Pfizer; has received grants from Novartis; has received grants and non-financial support from AstraZeneca; and has received grants from Roche Genentech, Lilly, Orion, Amgen, Sanofi, Exact Sciences, Servier, MSD, BMS, Pierre Fabre, Besins, the European Commission, the French government, Fondation ARC, Taiho, and Elsan, outside the submitted work. BP has served in a consulting/advisory role for Puma Biotechnology, Novartis, Myriad Genetics, and Pierre Fabre; has received personal fees from Novartis, AstraZeneca, MSD Oncology, and Pfizer; and has received research funding from Daiichi Sankyo, Puma Biotechnology, Novartis, Merus, Pfizer, and AstraZeneca. FA has received institutional research funding from AstraZeneca, Novartis, Pfizer, Eli Lilly, Roche, and Daiichi Sankyo; and has received travel, accommodations, and expenses support from Novartis, Roche, GlaxoSmithKline, and AstraZeneca. ER has received research support (institutional) from Gilead, MSD, and Menarini; has received honoraria from Eli Lilly, Seagen, Novartis, AstraZeneca, Daiichi Sankyo, MSD, Menarini, and Roche; has received travel, accommodations, and expenses support from Pfizer, Roche, Eli Lilly, Gilead, Novartis, Menarini, MSD, and Mundipharma; has served in a consultancy role for AstraZeneca, Novartis, and MSD; and has served on an advisory board for Gilead. All other authors declare no competing interests.

## Data Availability

Data are available upon reasonable written request to the corresponding author and approval of the STING study steering committee.
