# Supplementary material for: Genomic landscape of metastatic breast cancers in young adults: a liquid biopsy analysis of women aged 20–40 years
Source: Breast. 2026 Jan 2;85:104690. doi: 10.1016/j.breast.2025.104690 (PMC12818159; doi:10.1016/j.breast.2025.104690)
Supplement: Multimedia component 1 [file mmc1.docx]

| **Supplementary Table 1: ESCAT classification of breast cancer and agnostic targetable alterations** | | | | | | |
| --- | --- | --- | --- | --- | --- | --- |
| **Gene/Signature** | **Alteration** | **Estimated Prevalence** | **ESCAT Level** | **Drug Class** | **Tumor Type** | **References** |
| *NTRK1/2/3* | Fusions | Rare, varies by tumor type | I-C | TRK inhibitors | Tumor-agnostic | [1] |
| *MSI-H/dMMR* | Microsatellite instability-high  Mismatch repair deficient | Varies (~5% across cancers) | I-C | Checkpoint inhibitors | Tumor-agnostic | [1] |
| *RET* | Fusions | Rare (thyroid/salivary gland cancers) | I-C | RET inhibitors | Tumor-agnostic | [1] |
| *BRAF* | Mutations | Varies (40%-45% melanoma) | I-C | BRAF + MEK inhibitors | Tumor-agnostic | [1] |
| *FGFR1/2/3* | Fusions/Mutations | Varies by cancer type (10-40% bladder cancer) | I-C | Pan-FGFR TKIs | Tumor-agnostic | [1] |
| TMB-H | High tumor mutation burden | Varies, high in lung cancer (40%) | I-C | Immune checkpoint inhibitors | Tumor-agnostic | [1] |
| *ERBB2 (HER2)* | Amplifications, Hotspot mutations | 15%-20% amplifications,  4% mutations | I-A (Amplifications),  II-B (Mutations) | Anti-HER2 ADCs/HER2 TKIs | Advanced BC | [2–5]  [6–8] |
| *PIK3CA* | Hotspot mutations | 30%-40% | I-A | PI3K inhibitors | Advanced BC (ER-positive, HER2-negative) | [9–11] |
| *ESR1* | Mutations | 30%-40% | I-A | Selective estrogen receptor degrader | Advanced BC (ER-positive, HER2-negative) | [12,13] |
| *BRCA1/2* | Germline pathogenic/likely pathogenic variants,  Somatic mutations | 4%  3% | I-A  II-B | PARP inhibitors | Advanced BC | [14–16]  [17] |
| *PTEN* | Mutations/deletions | 7 % | I/II | AKT inhibitors | Advanced BC | [9,18] |
| *AKT1* | Mutations | 5 % | I/II | AKT inhibitors | Advanced BC | [9,19] |
| *PALB2* | Germline pathogenic variants | 1% | II-B | PARP inhibitors | Advanced BC | [17,20] |

**References**

[1] Mosele MF, Westphalen CB, Stenzinger A, Barlesi F, Bayle A, Bièche I, et al. Recommendations for the use of next-generation sequencing (NGS) for patients with advanced cancer in 2024: a report from the ESMO Precision Medicine Working Group. Annals of Oncology 2024;35:588–606. https://doi.org/10.1016/j.annonc.2024.04.005.

[2] Rugo HS, Im S-A, Cardoso F, Cortés J, Curigliano G, Musolino A, et al. Efficacy of Margetuximab vs Trastuzumab in Patients With Pretreated ERBB2-Positive Advanced Breast Cancer: A Phase 3 Randomized Clinical Trial. JAMA Oncology 2021;7:573–84. https://doi.org/10.1001/jamaoncol.2020.7932.

[3] Krop IE, Kim S-B, González-Martín A, LoRusso PM, Ferrero J-M, Smitt M, et al. Trastuzumab emtansine versus treatment of physician’s choice for pretreated HER2-positive advanced breast cancer (TH3RESA): a randomised, open-label, phase 3 trial. The Lancet Oncology 2014;15:689–99. https://doi.org/10.1016/S1470-2045(14)70178-0.

[4] Lin NU, Borges V, Anders C, Murthy RK, Paplomata E, Hamilton E, et al. Intracranial Efficacy and Survival With Tucatinib Plus Trastuzumab and Capecitabine for Previously Treated HER2-Positive Breast Cancer With Brain Metastases in the HER2CLIMB Trial. JCO 2020;38:2610–9. https://doi.org/10.1200/JCO.20.00775.

[5] Saura C, Oliveira M, Feng Y-H, Dai M-S, Chen S-W, Hurvitz SA, et al. Neratinib Plus Capecitabine Versus Lapatinib Plus Capecitabine in HER2-Positive Metastatic Breast Cancer Previously Treated With ≥ 2 HER2-Directed Regimens: Phase III NALA Trial. JCO 2020;38:3138–49. https://doi.org/10.1200/JCO.20.00147.

[6] Li BT, Meric-Bernstam F, Bardia A, Naito Y, Siena S, Aftimos PG, et al. 654O Efficacy and safety of trastuzumab deruxtecan (T-DXd) in patients (pts) with solid tumors harboring specific HER2-activating mutations (HER2m): Primary results from the international phase II DESTINY-PanTumor01 (DPT-01) study. Annals of Oncology 2023;34:S459–60. https://doi.org/10.1016/j.annonc.2023.09.1840.

[7] Hyman DM, Piha-Paul SA, Won H, Rodon J, Saura C, Shapiro GI, et al. HER kinase inhibition in patients with HER2- and HER3-mutant cancers. Nature 2018;554:189–94. https://doi.org/10.1038/nature25475.

[8] Smyth LM, Piha-Paul SA, Won HH, Schram AM, Saura C, Loi S, et al. Efficacy and Determinants of Response to HER Kinase Inhibition in HER2-Mutant Metastatic Breast Cancer. Cancer Discovery 2020;10:198–213. https://doi.org/10.1158/2159-8290.CD-19-0966.

[9] Turner NC, Oliveira M, Howell SJ, Dalenc F, Cortes J, Moreno HLG, et al. Capivasertib in Hormone Receptor–Positive Advanced Breast Cancer. New England Journal of Medicine 2023;388:2058–70. https://doi.org/10.1056/NEJMoa2214131.

[10] Rugo HS, Lerebours F, Ciruelos E, Drullinsky P, Ruiz-Borrego M, Neven P, et al. RETRACTED: Alpelisib plus fulvestrant in *PIK3CA*-mutated, hormone receptor-positive advanced breast cancer after a CDK4/6 inhibitor (BYLieve): one cohort of a phase 2, multicentre, open-label, non-comparative study. The Lancet Oncology 2021;22:489–98. https://doi.org/10.1016/S1470-2045(21)00034-6.

[11] André F, Ciruelos E, Rubovszky G, Campone M, Loibl S, Rugo HS, et al. Alpelisib for PIK3CA-Mutated, Hormone Receptor–Positive Advanced Breast Cancer. New England Journal of Medicine 2019;380:1929–40. https://doi.org/10.1056/NEJMoa1813904.

[12] Bardia A, Bidard F-C, Neven P, Streich G, Montero AJ, Forget F, et al. Abstract GS3-01: GS3-01 EMERALD phase 3 trial of elacestrant versus standard of care endocrine therapy in patients with ER+/HER2- metastatic breast cancer: Updated results by duration of prior CDK4/6i in metastatic setting. Cancer Research 2023;83:GS3-01. https://doi.org/10.1158/1538-7445.SABCS22-GS3-01.

[13] Bidard F-C, Kaklamani VG, Neven P, Streich G, Montero AJ, Forget F, et al. Elacestrant (oral selective estrogen receptor degrader) Versus Standard Endocrine Therapy for Estrogen Receptor–Positive, Human Epidermal Growth Factor Receptor 2–Negative Advanced Breast Cancer: Results From the Randomized Phase III EMERALD Trial. JCO 2022;40:3246–56. https://doi.org/10.1200/JCO.22.00338.

[14] Tutt ANJ, Garber JE, Kaufman B, Viale G, Fumagalli D, Rastogi P, et al. Adjuvant Olaparib for Patients with BRCA1- or BRCA2-Mutated Breast Cancer. New England Journal of Medicine 2021;384:2394–405. https://doi.org/10.1056/NEJMoa2105215.

[15] Robson ME, Im S-A, Senkus E, Xu B, Domchek SM, Masuda N, et al. OlympiAD extended follow-up for overall survival and safety: Olaparib versus chemotherapy treatment of physician’s choice in patients with a germline BRCA mutation and HER2-negative metastatic breast cancer. European Journal of Cancer 2023;184:39–47. https://doi.org/10.1016/j.ejca.2023.01.031.

[16] Litton JK, Rugo HS, Ettl J, Hurvitz SA, Gonçalves A, Lee K-H, et al. Talazoparib in Patients with Advanced Breast Cancer and a Germline BRCA Mutation. New England Journal of Medicine 2018;379:753–63. https://doi.org/10.1056/NEJMoa1802905.

[17] Tung NM, Robson ME, Ventz S, Santa-Maria CA, Nanda R, Marcom PK, et al. TBCRC 048: Phase II Study of Olaparib for Metastatic Breast Cancer and Mutations in Homologous Recombination-Related Genes. JCO 2020;38:4274–82. https://doi.org/10.1200/JCO.20.02151.

[18] Schmid P, Abraham J, Chan S, Wheatley D, Brunt AM, Nemsadze G, et al. Capivasertib Plus Paclitaxel Versus Placebo Plus Paclitaxel As First-Line Therapy for Metastatic Triple-Negative Breast Cancer: The PAKT Trial. JCO 2020;38:423–33. https://doi.org/10.1200/JCO.19.00368.

[19] Kalinsky K, Hong F, McCourt CK, Sachdev JC, Mitchell EP, Zwiebel JA, et al. Effect of Capivasertib in Patients With an AKT1 E17K-Mutated Tumor: NCI-MATCH Subprotocol EAY131-Y Nonrandomized Trial. JAMA Oncology 2021;7:271–8. https://doi.org/10.1001/jamaoncol.2020.6741.

[20] Gruber JJ, Afghahi A, Timms K, DeWees A, Gross W, Aushev VN, et al. A phase II study of talazoparib monotherapy in patients with wild-type BRCA1 and BRCA2 with a mutation in other homologous recombination genes. Nat Cancer 2022;3:1181–91. https://doi.org/10.1038/s43018-022-00439-1.
